# Model-Driven Redox Pathway Manipulation for Improved Isobutanol Production in *Bacillus subtilis* Complemented with Experimental Validation and Metabolic Profiling Analysis

**DOI:** 10.1371/journal.pone.0093815

**Published:** 2014-04-04

**Authors:** Haishan Qi, Shanshan Li, Sumin Zhao, Di Huang, Menglei Xia, Jianping Wen

**Affiliations:** 1 Key Laboratory of System Bioengineering, Ministry of Education, Tianjin University, Tianjin, People's Republic of China; 2 Department of Biochemical Engineering, School of Chemical Engineering and Technology, Tianjin University, Tianjin, People's Republic of China; 3 Collaborative Innovation Center of Chemical Science and Engineering, Tianjin, People's Republic of China; University of Nebraska-Lincoln, United States of America

## Abstract

To rationally guide the improvement of isobutanol production, metabolic network and metabolic profiling analysis were performed to provide global and profound insights into cell metabolism of isobutanol-producing *Bacillus subtilis*. The metabolic flux distribution of strains with different isobutanol production capacity (BSUL03, BSUL04 and BSUL05) drops a hint of the importance of NADPH on isobutanol biosynthesis. Therefore, the redox pathways were redesigned in this study. To increase NADPH concentration, glucose-6-phosphate isomerase was inactivated (BSUL06) and glucose-6-phosphate dehydrogenase was overexpressed (BSUL07) successively. As expected, NADPH pool size in BSUL07 was 4.4-fold higher than that in parental strain BSUL05. However, cell growth, isobutanol yield and production were decreased by 46%, 22%, and 80%, respectively. Metabolic profiling analysis suggested that the severely imbalanced redox status might be the primary reason. To solve this problem, gene *udhA* of *Escherichia coli* encoding transhydrogenase was further overexpressed (BSUL08), which not only well balanced the cellular ratio of NAD(P)H/NAD(P)^+^, but also increased NADH and ATP concentration. In addition, a straightforward engineering approach for improving NADPH concentrations was employed in BSUL05 by overexpressing exogenous gene *pntAB* and obtained BSUL09. The performance for isobutanol production by BSUL09 was poorer than BSUL08 but better than other engineered strains. Furthermore, in fed-batch fermentation the isobutanol production and yield of BSUL08 increased by 11% and 19%, up to the value of 6.12 g/L and 0.37 C-mol isobutanol/C-mol glucose (63% of the theoretical value), respectively, compared with parental strain BSUL05. These results demonstrated that model-driven complemented with metabolic profiling analysis could serve as a useful approach in the strain improvement for higher bio-productivity in further application.

## Introduction

Isobutanol, an important platform compound in food, pharmaceutical and chemical industry has received significant attention [1]. Especially, as ideal gasoline additives or substitutes, isobutanol has higher energy density and octane number, and lower hygroscopicity compared with traditional biofuels [2], [3].

Owing to its importance for biofuels and its broad applicability, considerable efforts have been made to enhance the production of isobutanol. Among them, some strains had been engineered as cell factory for isobutanol production using synthetic biology and metabolic engineering, such as *Escherichia coli* [2], [4], [5], *Corynebacterium glutamicum* [6], *Saccharomyces cerevisiae* [7] and *Clostridium cellulolyticum* [8], etc. In addition, isobutanol-producing *Bacillus subtilis* was also engineered for its relatively high solvent tolerance in our previous work, and the titer of isobutanol by engineered *B. subtilis* BSUL03 was 2.62 g/L in unbaffled shake-flask fed-batch fermentation [9]. To achieve the efficient strain improvement, the genome-scale metabolic network of BSUL03 was constructed and analyzed by elementary mode analysis for the first time. In two-stage fed-batch fermentation, the mutant BSUL 05 (Δ*ldh* and Δ*pdhC*) produced 5.5 g/L isobutanol [10].

Compared with other isobutanol producer [2], the mutant BSUL 05 still need further optimization. In our previous work, 6 target genes related to the pathways of reducing power were predicted except pyruvic acid branch pathway and α-ketoisovalerate biosynthetic pathway which had been engineered [9], [10]. The redox status of cell affects a broad range of genes expression and cellular functions, as well as metabolite profiles. It plays crucial roles in the metabolic and regulatory network of living organisms [11], [12], [13]. In addition, the ketol-acid reductoisomerase and alcohol dehydrogenase for isobutanol biosynthesis are NADPH-dependent and NADH-dependent, respectively, in BSUL05. One equivalent of NADPH and one equivalent of NADH are required for conversion of pyruvate to isobutanol. So the improvement of redox status may be an important direction for BSUL 05 improvement. In fact, some approaches have been reported to improve isobutanol production by optimizing NAD(P)H availability, such as the improvement of NADPH availability by overexpressing the transhydrogenase gene *pntAB* from *E. coli*, which was proved to be highly beneficial for isobutanol production in *E. coli* or *C. glutamicum* [6], [14], the synergistic effect of transhydrogenase PntAB and NAD kinase YfjB on increasing NADPH supply and improving anaerobic isobutanol production [15]. So far, the systematic proof about the importance of redox status has not been put forward in *B. subtilis* for isobutanol biosynthesis. Since redox cofactors NADPH and NADH are widespread cofactors that participate in more than 100 reactions [16], it is necessary to establish a rational method for the engineering of efficient redox pathway.

Here, the great importance of cellular redox status for isobutanol biosynthesis was demonstrated systematically by the metabolic flux distribution and metabolic profiling analysis in *B. subtilis*. Furthermore, the redox pathway was engineered by inactivating glucose-6-phosphate isomerase (PGI), overexpressing glucose-6-phosphate dehydrogenase (G6PD) and transhydrogenases (UdhA and PntAB), which successfully improved isobutanol production.

## Materials and Methods

### Reagents

All enzymes were purchased from Fermentas Co., Ltd (Glen Burnie, MD, USA). All antibiotics were purchased from Sigma-Aldrich (St. Louis, MO, USA). Oligonucleotides were supplied by Invitrogen Biotechnology Co., Ltd (Carlsbad, CA, USA), and DNA sequencing was served by BGI (Beijing, China). Reagents used for MS samples preparation were all purchased from Sigma or Fluka except pyridine (Aladdin, China). Solvents used for chromatography were LC-MS grade. High purity solvents and reagents were used in order to avoid appearances of interfering MS peaks and high background. Water was deionized and filtered through 0.22 μm filters by a Millipore water generation system (Millipore, Schwalbach, Germany).

### Bacterial strains, plasmids, and genetic manipulation

The strains and plasmids used in this work are listed in Table 1. All *B. subtilis* mutants used in this work were the derivatives of *B. subtilis* 168 which had been engineered in our previous report [10]. *E. coli* JM109 was used to propagate all plasmids. All oligonucleotides used in this work are listed in Table 2. Plasmid pRPCmP02 was constructed for gene knockout of *pgi*. The up- and down-stream homologous arms *pgi*-I and *pgi*-II were amplified with two pairs of primers *pgi*-I-F and *pgi*-I-R, *pgi*-II-F and *pgi*-II-R, respectively, using genomic DNA of BSUL05 as template. The PCR product of *pgi*-I was digested with *Nde*I-*Pst*I, and then cloned into plasmid pRPCm cut with the same enzymes, creating plasmid pRPCmP01. Similarly, the *Bgl*II-*EcoR*I digested *pgi*-II PCR product was inserted into pRPCmP01 cut with the same enzymes, generating plasmid pRPCmP02. To overexpress G6PD, plasmid pRPCmPZT was constructed. To minimize the influence of knock-in of *zwf* on the downstream genes expression, a strong terminator T0T1T2 was added. Gene *zwf* encoding G6PD was amplified from genomic DNA of *B. subtilis* by using a pair of primers *zwf*-F and *zwf*-R, while the strong terminator was obtained from plasmid pMUTIN4 with a pair of primers Ter-F and Ter-R. The overlapped PCR product of *zwf*-T0T1T2 was generated by using the purified *zwf* and T0T1T2 PCR products mixture as template with a pair of primers *zwf*-F and Ter-R. Further, the *zwf*-T0T1T2 PCR was digested with *Kpn*I-*Bgl*II, and then cloned into plasmid pRPCmP02 cut with the same enzymes, constructing plasmid pRPCmPZT. In order to overexpress transhydrogenase UhdA, plasmid pRPCmPZTU was designed. The heterologous transhydrogenase encoding gene *udhA* was obtained from *E. coli* BW25113 with a pair of primers *udh*A-F and *udhA*-R. The *udhA* PCR product was treated with *Kpn*I-*BamH*I and then inserted into plasmid pRPCmPZT digested with the same enzymes, creating plasmid pRPCmPZTU. To overexpress transhydrogenase PntAB, plasmid pRPCmPAB was constructed. The heterologous transhydrogenase encoding gene *pntAB* was obtained from *E. coli* MG1655 with primer set *pntAB*-F and *pntAB*-R. The *pntAB* PCR product was digested with *BamH*I and *Pst*I, and cloned into plasmid pRPCm at *BamH*I and *Pst*I sites, resulting in pRPCmPAB.

**Table 1:** Strains and plasmids used in this work.

Name	Relevant genotype	Source
**Strains**		
*E. coli* JM109	*recA*1, *endA*1, *gyrA*96, *thi*-1, *hsdR*17, *supE*44, *relA*1, Δ(*lac*-*proAB*)/*F'*[*traD*36, *proAB+*, *lacIq*, *lacZ*Δ*M*15]	TransGen Biotech
*E. coli* BW25113	*lacIq*, *rrnBT*14, Δ*lacZWJ*16, *hsdR*514, Δ*araBADAH*33, Δ*rhaBADLD*78	CGSC^a^
*B.subtilis* 168	Wide-type strain, *trpC2*	BGSC^b^
BSUL03	Δ*amyE*::(P_43_::*kivd*-*adh2*), P_43_::*ilvD*-*ilvC*-*alsS*; Spc^r^, Em^r^	[9]
BSUL04	BSUL03 with gene *ldh* disruption; Spc^r^, Em^r^, Km^r^	[10]
BSUL05	BSUL03 with gene *ldh*, *pdhC* disruption; Spc^r^, Em^r^, Km^r^, Tet^r^	[10]
BSUL06	BSUL05 with gene *pgi* disruption	This work
BSUL07	BSUL06 with gene *zwf* overexpression	This work
BSUL08	BSUL07 with heterologous gene *udhA* overexpression	This work
BSUL09	BSUL05 with heterologous gene *pntAB* overexpression	This work
**Plasmids**		
pMUTIN4	*B. subtilis* integration plasmids; Cm^r^, Amp^r^	BGSC^b^
pRPCm	*B. subtilis* integration plasmids derived from pUC18, P_43_::; Cm^r^, Amp^r^	Laboratory stock
pRPCmP01	pRPCm with upstream homologous fragment of gene *pgi*	This work
pRPCmP02	pRPCmP01 with downstream homologous fragment of gene *pgi*	This work
pRPCmPZT	pRPCmP02 with the fusion fragment of glucose-6-phosphate 1-dehydrogenase gene *zwf* and T0-T1T2 terminator	This work
pRPCmPZTU	pRPCmPZT with the transhydrogenase gene *udhA* of *E. coli* BW25113	This work
pRPCmPAB	pRPCm with the transhydrogenase gene *pntAB* of *E. coli* MG1655	This work

a CGSC: Coli Gentic Stock Center.

b BGSC: Bacillus Gentic Stock Center.

**Table 2:** Oligonucleotides used in this study.

Primer	Sequence (5′→3′)
*pgi*-I-F	TGCTACATATGACTTCGGTGACTGGGCT
*pgi*-I-R	CTTATCATATGCTGCAGCAAAGCGTACATGCGTC
*pgi*-II-F	TGATTAGATCTCGCGATGAGCGGTTAC
*pgi*-II-R	CGCCGGAATTCATTAAGAAGTAAACAGCC
*zwf*-F	CTATAGGTACCTCGAGAATGGATCCTAAAATAAAGCTTCGA
*zwf*-R	GTGTAACTTTCCAAATTTACGCGGTTATATGTTCCACCAGTGTAAGC
Ter-F	GCTTACACTGGTGGAACATATAACCGCGTAAATTTGGAAAGTTACAC
Ter-R	GAGTGAGATCTGTGCCGAATAGTCTGGACTG
*udhA*-F	CTAGTGGTACCTGCCATAGTAATAGGTTCCC
*udhA*-R	CTCACGGATCCAAGAATGGATGGCCATTTC
*pntAB*-F	GCATGGATCCAAGGAGATATACCATGCGAATTGGCATACCAAGAG
*pntAB*-R	GCATCTGCAGTTACAGAGCTTTCAGGATTGCATC

The PGI-deficient mutant (BSUL06), G6PD-overexpressed mutant (BSUL07) and transhydrogenase-overexpressed mutant (BSUL08 and BSUL09) were obtained by transforming plasmids pRPCmP02, pRPCmPZT, pRPCmPZTU and pRPCmPAB into BSUL05 using the competent cell method [17], respectively. The positive recombinants were selected by chloramphenicol resistance, and further confirmations were performed by PCR of *pgi*-deficient mutant BSUL06, *zwf*-overexpression strain BSUL07, *udhA*-overexpression strain BSUL08 and *pntAB*-overexpression strain BSUL09 using primers *pgi*-I-F/*pgi*-II-R, *zwf*-F/Ter-R, *udhA*-F/*udhA*-R, and *pntAB*-F/*pntAB*-R, respectively, which were listed in Table 2.

### Medium and cultivation conditions

Unless stated otherwise, *E. coli* and *B. subtilis* were cultured in Luria-Bertani (LB) medium (peptone 10 g/L, yeast extract 5 g/L, and sodium chloride 5 g/L) at 37°C. Batch fermentation was carried out in LBGSM-I medium (LB medium with glucose 20 g/L, K_2_HPO_4_ 2 g/L, KH_2_PO_4_ 1 g/L and 10^3^ dilution of Trace Metal Mix A5 [2]). Fed-batch fermentation was performed in LBGSM-III medium (identical to LBGSM-I except for glucose 10 g/L). Glucose feeding solution (glucose 500 g/L) and acetate feeding solution (sodium acetic acid 200 g/L) were used as feeding stocks during fed-batch fermentation. For all engineered strains, sodium acetic acid was originally supplemented into the medium at a final concentration of 3 g/L and 3.4 g/L in batch and fed-batch fermentation, respectively. Antibiotics were added appropriately as follows: ampicillin 100 μg/mL, chloramphenicol 50 and 5 μg/mL for *E. coli* and *B. subtilis*, respectively. Seed cultures were prepared by inoculating one fresh colony into liquid LB medium and cultivated at 240 rpm for 8 h. The 1% inoculation was adopted in all experiments. For phenotype growth and metabolic profiling analysis, cells were cultured in LBGSM-I medium in 500 mL screw-cap flasks for 40 h (40% work volume, 200 rpm, 37°C). For fermentation, cells were cultivated in 400 mL LBGSM-III medium in a fed-batch fermentation system (DASGIP, Germany) under two-stage (aerobic/oxygen-limited) conditions for 60 h as described in our previous work [10]. All the experiments were performed in three biological replicates.

### Modeling of metabolic network

**Figure 1:**
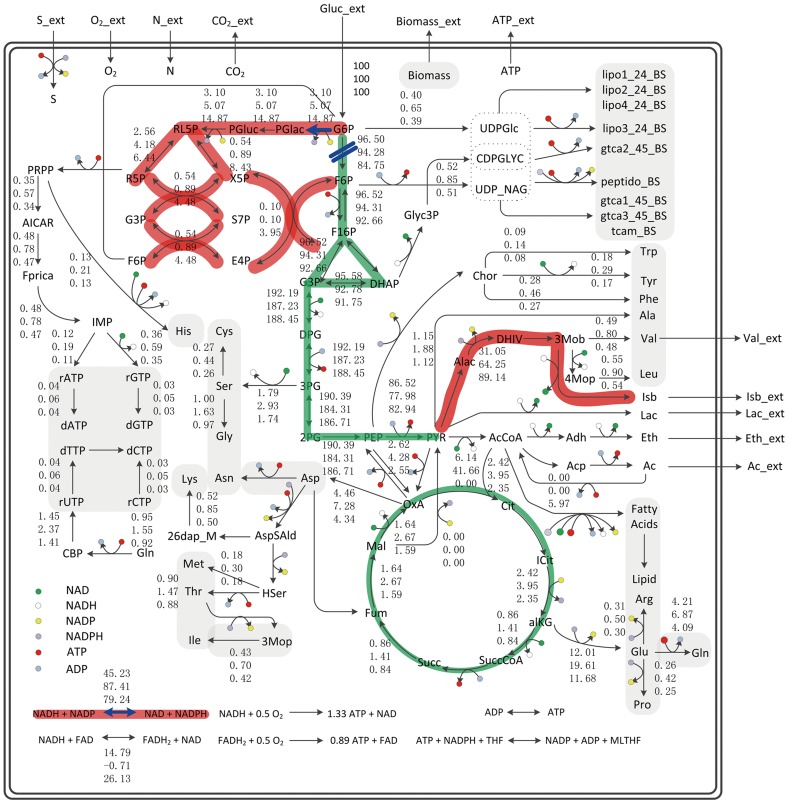
The metabolic flux distribution in isobutanol-producing strain *B. subtilis*. Data represented *in silico* flux distribution of different isobutanol-producing strains (top BSUL03, middle BSUL04, down BSUL05). Bold red and green lines represented the increased and decreased flux, respectively. The blue marks represented the targets for redox pathway engineering in this work. Abbreviations were listed in previous work [10]. Part of the flux data in central metabolism were taken from previous work [10].

The genome-scale metabolic network of isobutanol-producing *B. subtilis* was constructed and further refined for analysis in our previous work [10]. It includes glycolysis pathway (EMP), pentose phosphate pathway (PPP), tricarboxylic acid (TCA) cycle, biosynthesis pathway, transportation, anaplerosis and respiratory chain as depicted in Figure 1. Here the intracellular metabolism was elucidated by EMA, which was implemented by METATOOL 5.1 (http://www.biozentrum.uni-wuerzburg.de/bioinformatik/) [18]. EMA results were analyzed with Excel Microsoft Corp. for mode sorting and filtering.

### Enzymatic activity assays in crude extracts

Cells cultivated for enzyme assays were grown at 37°C in 25 mL LB medium and harvested at the exponential phase (6 h). The preparation of crude cell extracts was carried out as described previously [10]. Activity of PGI was determined by observing the absorbance variation of NADH at 340 nm at 25°C. Activities of G6PD and transhydrogenases (UdhA and PntAB) were determined spectrophotometrically at 30°C by measuring the changes in A_340_ and A_375_ of NADPH, respectively. All reaction systems were fixed at 1 mL. PGI assay system contains 100 mM Tris-HCl (pH 7.6), 0.5 mM NAD^+^, 1 U G6PD, 2 mM fructose-6-phosphate and 100 μL crude cell extracts. Reaction mixture for G6PD contains 100 mM Tris-HCl (pH 7.5), 200 mM KCl, 1 mM NADP^+^, 10 mM MgCl_2_, 5 mM glucose-6-phosphate (G6P) and 100 μL crude cell extracts [19]. Reaction mixture for UdhA and PntAB includes 50 mM Tris-HCl (pH 7.6), 2 mM MgCl_2_, 0.5 mM NADPH, 1 mM 3-acetylpyridine adenine dinucleotide and 100 μL crude cell extracts [16].

### Determination of intracellular NAD(P)H, NAD(P)^+^, ADP and ATP

The determination of intracellular nucleotides, NAD(P)H, NAD(P)^+^, ADP and ATP was conducted as described by Luo et al. [20] with slight modifications. In brief, 5 mL cultures cultivated for 12 h (in exponential phase) were rapidly sprayed into a 50 mL tube with 15 mL pre-cold (−70°C) aqueous methanol solution (60%, v/v) to stop cell metabolism quickly. Then the mixture was centrifuged at 10,000×*g*, −20°C for 10 min (Sigma 3–30K, Germany). After removing the supernatant, 0.5 mL pre-cold (−20°C) methanol-water (60%, v/v) was used to resuspend the cell debris by vortexing. Subsequently, the sample was blended with 2 mL of 0.3 M KOH (dissolved in 25% ethanol) and kept at −80°C. After thawing, the mixture was neutralized with 40 μL of glacial acetic acid. After centrifugation (10,000×g, −20°C, 10 min), the supernatant was collected and filtered with a 0.22 μm filter, and stored at −80°C for further analysis. Intracellular nucleotides were measured by a HPLC system (Agilent 1100) coupled with an Agilent mass spectrometer (Agilent 6410). The chromatographic separation and MS detection conditions were performed as described before [20].

### Intracellular metabolites analysis

#### Quenching, extraction of intracellular metabolites

Cells were quenched and extracted as described by Villas-Boas et al. [21] with slight modifications. The 36 h cultures were swiftly transferred into −40°C pre-cold methanol-water (60%, v/v) with a volume ratio of 1∶4 (sample: quenching buffer), and kept at the same temperature in an ethylene glycol bath for maximum 1 min. Subsequently, the cells were collected by centrifugation at 3,000×g, −20°C for 5 min, and then immediately transferred into the mortar which was precooled with the liquid nitrogen. Then about 50 mL liquid nitrogen was immediately poured into the mortar and meanwhile cell was ground to the fine powder with the pestle, which was repeated for at least three times to fully break the cells. The powder (50 mg) was suspended quickly with 1 mL −40°C pre-cold methanol-water (50%, v/v) extraction buffer and mixed thoroughly. The mixture was frozen in liquid nitrogen and thawed for three times. After centrifugation (−20°C, 17,900×g for 10 min), the supernatant was transferred into a 1.5 mL centrifugal tube (pre-cold in -20°C), while the cell debris was resuspended with another 0.5 mL buffer for further extraction. Thus about 1.5 mL extract was totally obtained. For relative quantification, the internal standard (0.1 mg/mL succinic d4 acid, 50 μL) was added into 200 μL aliquots from extracts. The mixture was frozen at −80°C and then lyophilized (ALPHA 1-2LD PLUS, Christ, Germany). Three biological replicates were performed for each sample.

#### Sample derivatization

Sample derivatization was performed using two-stage chemical derivatization [22] with some modifications. An aliquot (50 μL) O-methylhydroxylamine solution in pyridine was added into each lyophilized sample and incubated in water bath at 37°C for 90 min. The samples were then derivatized by addition of 80 μL N-acetyl-N-(trimethylsilyl)-trifluoroacetamide (MSTFA) and incubation at 37°C for 90 min.

#### Data collection

The derivatized samples were analyzed by GC-MS. One microliter of sample was injected by the autosampler (Agilent 7683) into GC (Agilent 7890A) equipped with the DB-5MS capillary column (30 m×0.25 mm, 0.25 μm, J&W Scientific, Folsom, CA). The injector and detector temperature were maintained at 250°C. The oven temperature was kept at 70°C for 2 min, then increased to 290°C (5°C/min) and kept for 2 min. The interface temperature of GC-MS was set at 280°C. The column effluent was introduced into the ion source (250°C) of MS (5975C MSD System, Agilent) with helium at 1.0 mL/min. Ions were generated by a 70 eV electron beam at an ionization current of 40 μA. The masses were acquired in the range of m/z 25–650 at a speed of 2 spectra per second.

#### Statistical analysis

For hierarchical clustering and comparison analysis, the data of metabolites were performed by Cluster 3.0 and visualized with Java TreeView.

### Other analytical methods

#### Biomass and glucose determination

Cells growth was determined by measuring the optical density of culture broth at 600 nm (OD600). Biomass (DCW) was calculated by multiplying OD600 by a coefficient of 0.325 [9]. Glucose was measured with a bioanalyzer (SBA-40C, China).

## Results

### Metabolic flux distribution in isobutanol-producing strain *B. subtilis*

Three isobutanol-producing strains *B. subtilis* BSUL03, BSUL04 and BSUL05 with different isobutanol-producing capacities (BSUL05> BSUL04> BSUL03) were constructed in our previous research [10]. In addition, the in silico metabolic network of isobutanol-producing strain *B. subtilis* which had been constructed and verified was utilized to analyze the metabolic flux.

According to the flux distribution and shift of BSUL03, BSUL04 and BSUL05 shown in Figure 1, more than 80% of carbon flux flowed through EMP in these three strains, which meant that glucose was converted into pyruvate mainly through the EMP. Furthermore, the flux through PPP showed a dramatic increase with the improvement of isobutanol-producing capacity. The flux of PPP in BSUL05 was 1.9 times and 3.8 times higher than that in BSUL04 and BSUL03, respectively. Moreover, the flux of G6P downstream reactions involved in EMP was decreased (Figure 1). As G6PD competed with PGI for the flux drained off G6P, the enervated EMP suggested that the cellular dynamic flux was adjusted to strengthen PPP. Since PPP is the major source for NADPH generation, it is speculated that NADPH plays an important role for isobutanol biosynthesis. Besides, in BSUL05, metabolic flux through the reaction responsible for redox balance was about 75% higher than that in BSUL03, but 9.3% lower than that in BSUL04. The function of redox balance for isobutanol biosynthesis need to be further investigated in engineered *B. subtilis*. According to the flux model analysis, redox cofactor was revealed to be a great potential limiting factor for isobutanol biosynthesis in BSUL05. For validation, it is imperative to implement experiments.

### Strategies for improving intracellular NADPH pool size

To settle the potential limiting factor of isobutanol biosynthesis in engineered *B. subtilis*, the deletion of PGI encoding gene *pgi*, as well as the overexpression of G6PD encoding gene *zwf* were implemented successively in BSUL05 for higher intracellular NADPH concentration.

**Figure 2:**
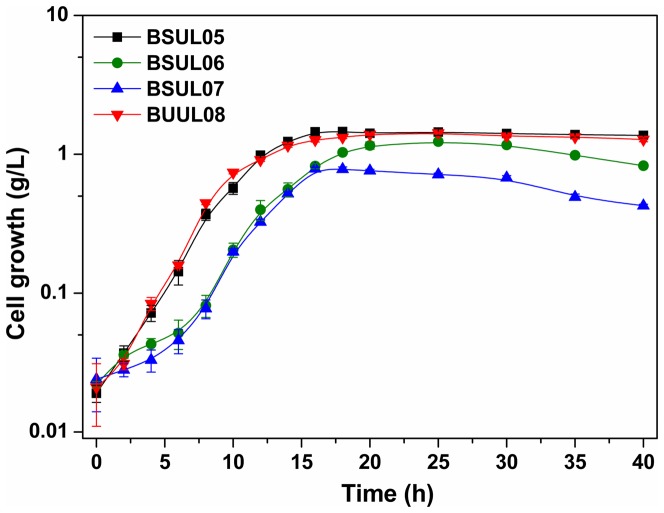
Comparison of cell growth profiles for different isobutanol-producing *B. subtilis*. The experiments were carried out in LBGSM-I medium under microaerobic conditions. Strains were cultivated in the medium supplemented with 3 g/L sodium acetic acid. Data were expressed as average values and standard deviations (SD) of three parallel studies.

**Figure 3:**
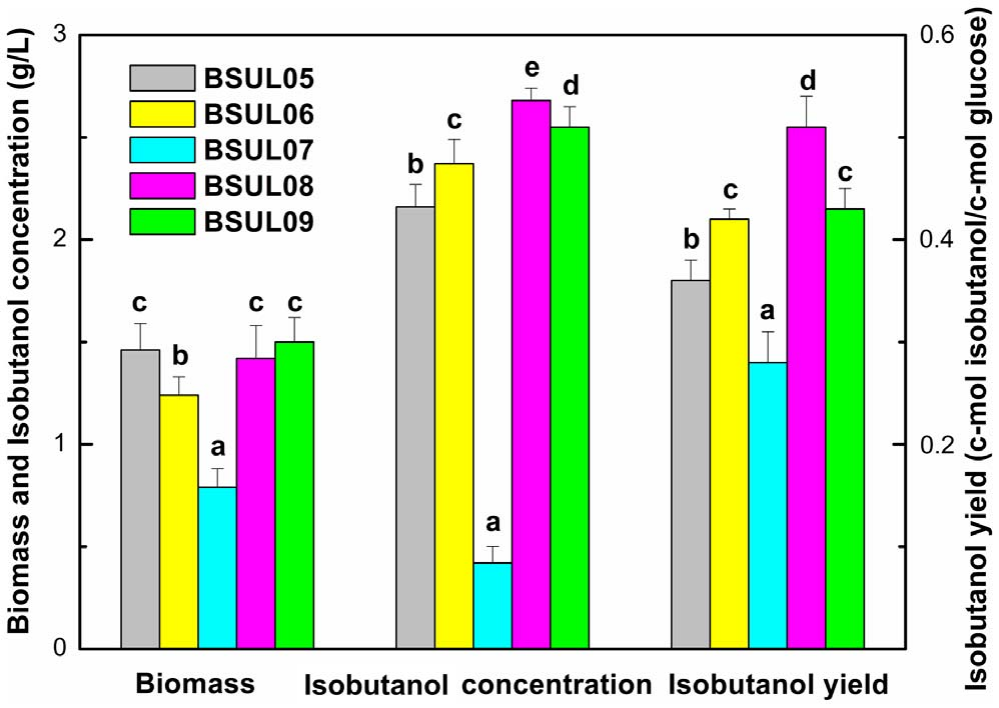
Biomass, isotutanol concentration and yield for different isobutanol-producing strains. Strains were cultivated in 500-I culture medium and supplemented with 3 g/L sodium acetic acid under microaerobic condition for 40 h. Data were expressed as average values and standard deviations (SD) of three parallel studies. Mean data accompanied by the same alphabet letters are not statistically significant at a level of p<0.05 (Tukey's Honestly Significant Difference test).

Gene *pgi* in BSUL05 was disrupted by integrating plasmid pRPCmP02 (Figure S1) into the chromosome. Recombinants resistant to chloramphenicol were selected for PCR confirmation by using a pair of primers *pgi*-I-F and *pgi*-II-R. BSUL05 had a 2.3 kb PCR band, while the *pgi*-disrupted strain BSUL06 showed a 2.5 kb PCR band (Figure S2). Only trace PGI activity was measured in BSUL06 relative to its parental strain BSUL05 (Table S1), indicating the absence of an active PGI enzyme. As expected, the knockout of *pgi* gene led to 118% increment for NADPH concentration (Table 3), and the isobutanol yield was increased by 16.7%, to 0.42 C-mol isobutanol/C-mol glucose with the isobutanol concentration reaching 2.37 g/L, which indicated that the elevated NADPH pool had a positive effect on isobutanol biosynthesis. However, the inactivation of PGI resulted in cell growth delay with the lag phase from 4 h for BSUL05 extending to 6 h for BSUL06 (Δ*pgi*). Additionally, cell growth peaked at 25 h, and decreased thereafter (Figure 2). Correspondingly, compared with BSUL05, the maximal cell growth for BSUL06 were slightly decreased by 15% (Figure 3).

**Table 3:** Concentration of intracellular redox cofactors and energy for different isobutanol-producing *B. subtilis* strains.

	Concentrations (μmol/g DCW)								
Strains	NAD^+^	NADH	NADP^+^	NADPH	ADP	ATP	NADH/NAD^+^	NADPH/NADP^+^	ATP/ADP
BSUL05	374±36	318±27	62±11	143±20	124±15	313±29	0.85	2.31	2.52
BSUL06	252±28	273±12	117±9	312±19	190±21	294±33	1.08	2.67	1.55
BSUL07	138±29	154±16	119±21	632±52	112±12	126±14	1.12	5.31	1.13
BSUL08	458±52	356±49	269±28	517±48	149±20	382±27	0.77	1.92	2.57
BSUL09	364±45	295±29	173±18	373±25	132±17	328±36	0.81	2.15	2.48

Data were expressed as average values and standard deviations (SD) of three parallel studies.

*zwf* gene was overexpressed based on BSUL06 (Figure S3). Recombinants resistant to chloramphenicol were selected for PCR confirmation by using a pair of primers *zwf*-F and Ter-R (Table 2). BSUL06 showed a 0 kb PCR band, while BSUL07 presented a 2 kb PCR band (Figure S4). G6PD activity in BSUL07 was 2.5-fold relative to that in BSUL06 (Table S1), implying that *zwf* was overexpressed in BSUL07 with *pgi* deficiency. The determination of intracellular NADPH indicated that *zwf* overexpression in BSUL07 doubled NADPH concentration to 632 μmol/g DCW compared to that in BSUL06 (Table 3). However, the yield and titer of isobutanol in BSUL07 were 78% and 20% of that in the parent strain BSUL05, respectively (Figure 3). Moreover, the maximal cell growth of BSUL07 was seriously depressed, dramatically decreasing to 0.79 g/L (Figure 3). The above phenomenon illustrated that the cell growth and isobutanol biosynthesis of BSUL07 were severely inhibited. In addition, the cellular redox status is crucial for cell growth [23]. An excess of NADPH repressing cell growth has been reported by Canoaco et al. [24], Lim et al. [25] and Shimizu [26], which was mainly due to the inhibiton of G6PD and citrate synthase in TCA cycle, reducing the glucose comsumption, especially in the PGI mutant strains. Thus, in BSUL07, the redox status may be an important factor for regulating the lessened isobutanol production and the reduced cell growth.

### Metabolic profiling analysis for isobutanol-producing strain *B. subtilis*

**Figure 4:**
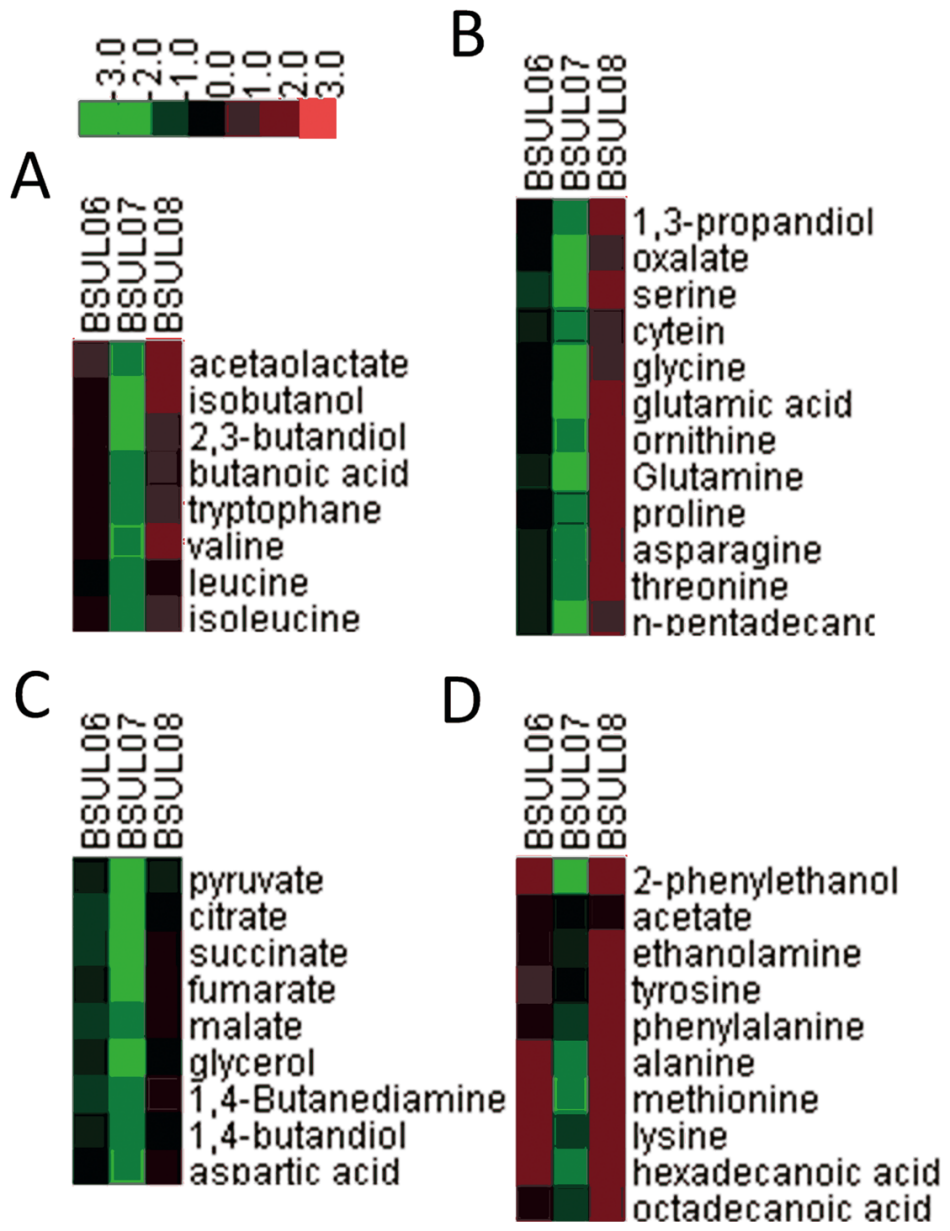
Fold changes of the major intracellular metabolites for different isobutanol-producing strains. The concentrations of different intracellular metabolites of reconstructed strains (BSUL06, BSUL07 and BSUL08) were normalized to that of BSUL05. Data presented in heat map were the average fold change values of each metabolite between the reconstructed strains and BSUL05.

To further evaluate the effect of cellular redox status on cell growth and isobutanol biosynthesis in isobutanol-producing strain *B. subtilis*, the intracellular metabolites, as well as nucleotides, NAD(P)H, NAD(P)^+^, ADP and ATP were detected and the results are shown in Figure 4 and Table 3, respectively.

As shown in Figure 4, a total of 39 metabolites were identified and classified into four clusters using hierarchical clustering and comparative analysis. Although cell growth was inhibited in *pgi*-deleted strain BSUL06 with slightly weak TCA cycle (citrate, succinate, fumarate, malate), the levels of metabolites involved in isobutanol biosynthetic pathway were increased, such as acetolactate (the precursor of isobutanol biosynthesis), which was in accordance with the improved isobutanol production (Figure 4). Moreover, the knockout of *pgi* gene led to the increment of NADPH and NADP^+^ concentration by 118% and 89%, respectively. Meanwhile the NADH level was decreased by approximately 14% (Table 3) due to the lower EMP activity, which was consistent with the previous reports [27], [28].

However, in *zwf* overexpressed mutant BSUL07 with *pgi* deletion, not only amino acids and fatty acid biosynthetic pathways, as well as TCA cycle were notably depressed, which further influenced the normal cell growth, but also isobutanol biosynthesis pathway was severely inhibited (Figure 4). The determination of intracellular redox cofactors suggested that NADPH concentration in BSUL07 was 4.4 times higher than that in BSUL05 and twice higher than that in BSUL06, respectively (Table 3). Whereas, the concentrations of ATP sharply dropped to 43% compared with that in BSUL06 (Table 3), which were in accord with the decreased biomass (Figure 2). Furthermore, the current NADPH/NADP^+^ was significantly shifted to 5.3, which was much higher than that in wild-type (1.84) [29], indicating that the redox balance was badly disrupted. The serious redox imbalance with excess NADPH could depress the oxidative PPP by inhibiting G6PD [30], which decreased intracellular levels of F6P and G3P and became an obstacle of downstream EMP and TCA cycle, resulting in the shortage of NADH and ATP for cell growth. These results implied that the redox imbalance was the primary factor that inhibited the cell growth and isobutanol biosynthesis in BSUL07.

### Improving redox balance through heterologous transhydrogenase overexpression

To ameliorate the current redox status, the exogenous gene *udhA* from *E. coli* encoding transhydrogenase UdhA was chosen to be overexpressed in BSUL08 (Figure S5). Recombinants resistant to chloramphenicol were selected for PCR confirmation by using a pair of primers *udhA*-F and *udhA* -R. BSUL05 had no PCR band, while BSUL08 showed a 1.4 kb PCR band (Figure S6). The UdhA activity of BSUL08 was 3.19-fold higher than that of BSUL07 (Table S1), implying that *udhA* was overexpressed with *pgi* deficient and *zwf* overexpression in BSUL08.

Although the NADPH concentration in BSUL08 decreased by 18% compared with BSUL07, it still was increased to 3.6-fold relative to that in BSUL05 (Table 3). Notably, compared with BSUL05 and BSUL07, NADPH/NADP^+^ in BSUL08 dropped by 16.5% and 64%, respectively, reaching 1.92 which was nearly equal to that in wildtype *B. subtilis* [29]. The drop of NADPH/NADP^+^ ratio suggests a depletion of the NADPH pool to suit isobutanol formation demand. Different with NADPH, intracellular NADH was the main source of ATP through oxidative phosphorylation, and the NADH/NAD^+^ should be in oxidation state (NADH/NAD^+^<1). Compared with BSUL05 and BSUL07, the rate of NADH/NAD^+^ in BSUL08 was decreased by 9% and 31%, respectively, reaching 0.78 which was within the appropriate ranges (0.5–0.8) in wildtype *B. subtilis* [31]. These data suggested that the redox balance was improved due to UdhA overexpression.

Furthermore, as depicted in Figure 2, cell growth for the *udhA*-overexpressed strain BSUL08 was well restored from the following aspects: (1) the lag phase was shortened to 2 h, which was similar to the parental strain BSUL05; (2) the specific cell growth rate was 83% higher than that in BSUL07; (3) the stationary phase was extended and the obvious cell lysis phenomenon disappeared; (4) the maximal biomass (1.42 g/L) was 80% higher than that in BSUL07. Meanwhile, the isobutanol yield and production were 42% and 24% higher than that in BSUL05, reaching 0.51 C-mol isobutanol/C-mol glucose and 2.68 g/L, respectivelly. In addition, the levels of metabolites involved in amino acids and fatty acids biosynthesis, TCA cycle and isobutanol biosynthesis were much higher than that in BSUL05 and BSUL07 (Figure 4). These results explained that the improvement of redox balance facilitated the cell growth and isobutanol biosynthesis in BSUL08, which further confirmed that the redox balance played an important role in cell growth and isobutanol biosynthesis.

In addition, based on the flux model analysis, a straightforward engineering approach for efficient NADPH availability was also carried out in BSUL05. The exogenous gene *pntAB* from *E. coli* encoding transhydrogenase PntAB, which transfers a hydride from NADH to NADP^+^ with the concurrent production of NADPH and NAD^+^, was overexpressed according to the description in Materials and Methods section, and BSUL09 was obtained. The PntAB activity of BSUL09 was 4.18-fold higher than that of BSUL05 (Table S1). The determination of intracellular NADPH indicated that *pntAB* overexpression in BSUL09 led to a 161% increment of NADPH concentration compared with that in BSUL05 (Table 3). The NADPH concentration in BSUL09 was lower than that in BSUL07 and BSUL08, but was increased by 20% than that in BSUL06. As expected, the isobutanol yield of BSUL09 was enhanced by 19.4% relatived to that of BSUL05, up to 0.43 C-mol isobutanol/C-mol glucose, which was close to that of BSUL06 and 84.3% of BSUL08 (Figure 3). The isobutanol production by BSUL09 was 2.55 g/L, 18% higher than that by BSUL05, which also demonstrated that the enhanced NADPH had a positive effect on isobutanol biosynthesis in BSUL05. For NADH, it showed a slight decline in BSUL09 compared with that in BSUL05, but its level was in the middle of that in BSUL08 and BSUL06/BSUL07 (Table 3). The ATP concentration in BSUL09 was higher than other strains except BSUL08, which also supported the higher biomass shown in Figure 3. Furthermore, compared with BSUL06 and BSUL07, NADPH/NADP^+^ in BSUL09 dropped by 19.5% and 59.5%, respectively, reaching 2.15, which was close to that in BSUL05 and BSUL09. And the ratio of NADH/NAP^+^ in BSUL09 had a similar trend compared with other engineered strains. These results illustrated that BSUL09 also had a well redox balance status, but the performance for isobutanol production by BSUL09 was poorer than BSUL08 and better than BSUL05, BSUL06 and BSUL07.

### Investigation of fermentation properties of BSUL08 in fed-batch fermentation

**Figure 5:**
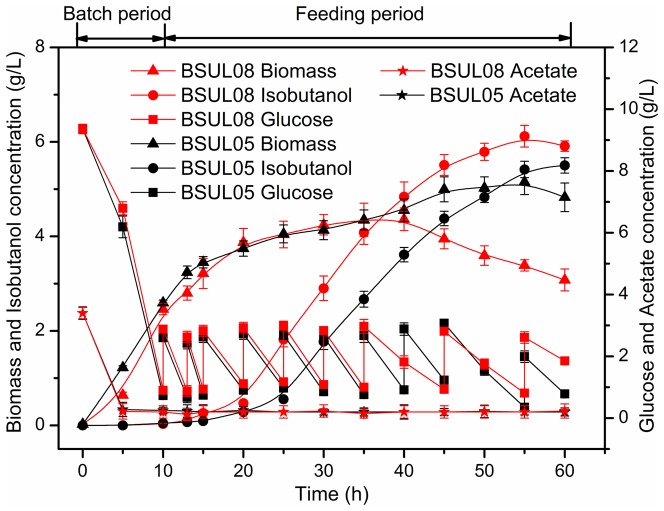
Fermentation properties of BSUL08 and BSUL05 in fed-batch fermentation. Data were expressed as average values and standard deviations (SD) of three parallel studies.

The investigation of genetic stability of engineered strain BSUL08 for ten generations demonstrated that BSLU08 exhibited good genetic stability for isobutanol production using the methods reported by Bi et al. [32]. To further boost isobutanol production, a fed-batch fermentation system under two-stage (aerobic/oxygen-limited) conditions as described in our previous work [10] was employed. Simultaneously, the parental strain BSUL05 as control was used, and the results are given in Figure 5. BSUL05 and its derivative strains were acetate auxotroph and required exogenous acetate as an additional carbon source for growth. Both strains grew exponentially under the aerobic conditions (batch period). During this period, strains displayed similar fermentation properties that glucose and acetate were almost exhausted with no isobutanol production. After entering the oxygen-limited period (feeding period), cell growth for both strains slowed down, whereas isobutanol biosynthesis began to be accelerated. Despite of 15% decrease of the maximal cell growth, isobutanol titer by BSUL08 was improved by 11%, reaching 6.12 g/L (Figure 5). Meanwhile, isobutanol yield was increased by 19%, up to 0.37 C-mol isobutanol/C-mol glucose, reaching 63% of the theoretical value [10]. The final acetate concentration of both strains was decreased to about 0.2 g/L. Furthermore, the cell growth of BSUL08 decreased obviously after 40 h fermentation, whereas it was much more stable for BSUL05. Based on the above results, it might be attributed to the cellular regulatory mechanisms of *B. subtilis* response to isobutanol, which was still unclear at present. In general, some similar alcohols such as ethanol and n-butanol are toxic for cell growth by disrupting cellular membrane, which is thought to occur by direct insertion of lipophilic side chain into the cellular membrane, destroying the protein-lipid interactions and inhibiting the glucose and nutrient transport [33], [34]. Besides, although the cell growth of BSUL08 was seriously inhibited, the isobutanol titer was increased continuously and reached the peak at 55 h. This phenomenon was also observed in *E. coli* by Atsumi et al. [2]. It may be due to the sustaining shortly cell free catalytic system which was composed of intracellular enzymes, cofactors and so on after cell death, and the system had been used to produce hydrogen in vitro [35].

## Discussion

Some progress had made in strain improvement of the engineered isobutanol-produceing *B. subtilis* in our group [9], [10], [36], [37]. However, the titier and the yield of the best producing strain BSUL05 was suboptimal and certainly improvable [10]. Since two isobutanol biosynthetic steps catalyzed by ketol-acid reductoisomerase and alcohol dehydrogenase require NAD(P)H as cofactors, therefore, NADPH and NADH are necessary for isobutanol biosynthesis and need to be well considered for strain improvement. The metabolic flux distribution analysis combined with metabolomics, which had been demonstrated to be an effective and powerful approach [38], was employed to improve isobutanol-producing strains.

The analysis of metabolic flux distribution based on the genome-scale network model in different engineered isobutanol-producing strains suggested that PPP had a positive correlation with isobutanol biosynthesis and the supply of NADPH may be crucial for efficient isobutanol biosynthesis in BSUL05, an engineered strain in our previous work [10]. In fact, some researchers had supposed that the lower isobutanol production could be relevant to the inadequate intracellular NADPH. Shi et al. improved the redox status to promote isobutanol biosynthesis by activating transhydrogenase and NAD kinase with NADPH supply increasement [15]. Besides, an NADH-dependent pathway was constructed to improve the utilization ability of the NADH, which enabled isobutanol to be biosynthesized by *E. coli* under anaerobic condition [14].

NADPH is generated mainly via oxidation of G6P by the PPP, which supplies reducing power for cellular biosynthetic processes [16]. Furthermore, several attempts have been made to increase the metabolic flux through the PPP by activating G6PD encoding gene *zwf* or inactivating PGI encoding gene *pgi* [11], [12], [25], [39], [40], [41]. However, the activity of the oxidative PPP is very low as indicated in Figure 1 because the EMP has evolved to dominate under natural conditions [42]. Therefore, the decrease in glycolytic activity should be coupled with activation of the PPP to increase NADPH concentration. Therefor, genes *pgi* and *zwf* were selected to engineer successively to increase NADPH concentration. The PGI disrupted strain BSUL06 displayed a higher NADPH level and isobutanol yield and an repressive cell growth compared with BSUL05. The increased NADPH could meet the requirements of NADPH as the cofactors in isobutanol biosynthetic step catalyzed by ketol-acid reductoisomerase, which could be the reason of improved isobutanol production with decreased biomass in BSUL06. In addition, Prasad and Freese [43] also observed the cell lysis phenomenon in PGI- and G6PD-deficient *B. subtilis*, and they ascribed this phenomenon to the intracellular accumulation of glucose-1-phosphate (G1P), which could inhibit the activities of enzymes responsible for the conversion of glucosamine-6-phosphate to *N*-acetyl glucosamine-1-phosphate and further inhibite the muramic acid pathway. The flux of G6P to G1P catalysed by phosphoglucomutase (PGM) may be increased in BSUL06, which was caused by PGI inactivation and a large amount of flux of G6P to EMP blocked. The knockout of *pgi* gene and overexpression of *zwf* gene in engineered strain BSUL07 made NADPH concentration be increased to double and 4.4-fold compared with that in BSUL06 and BSUL05, respectively (Table 3). However, the maximal isobutanol production and biomass of BSUL07 were seriously depressed (Figure 3). Due to the higher appetency of G6PD to G6P than PGM [43], the overexpressed *zwf* in BSUL07 could reduce the concentration of intracellular G1P. So the growth inhibition of BSUL07 may not be caused by G1P. Besides, It has been reported that excess NADPH could depress the cell growth [24], [25].

The metabolic profiling analysis involved in intracellular metabolites and redox cofators was performed to evaluate the effect of cellular redox status on the cell growth and isobutanol biosynthesis. The analysis of metabolite profiles suggested that NADPH/NADP^+^ was serious imbalance (Table 3) and amino acids and fatty acids biosynthetic pathways, as well as TCA cycle were notably inhibited in BSUL07 (Figure 4). Moreover, it was has been reported that the intracellular redox imbalance could significantly reduce the production of target products. The imbalance of redox cofactors was the major bottleneck for ethanol production by *S. cerevisiae* using pentose sugars [44]. Trinh et al. [45] reported that the imbalance of intracellular NADH/NAD^+^ was the main factor for no isobutanol production by *E. coli* under anaerobic condition. The *pntAB* transhydrogenase gene from *E. coli* was expressed in *C. glutamicum* resulted a more balanced redox status and tripled the isobutanol production [6]. An unregulated increase in the intracellular NADPH pool may result in the overexpression of the enzymes that maintain redox equivalents, thereby exerting burdens to cells to have a negative effect on the metabolism [27]. Thus, it was significant to maintain the redox balance and improve the NADPH level for isobutanol biosynthesis.

Previous investigations demonstrated that *B. subtilis* possesses the capacity to maintain the cellular redox balance [46]. However, the enzyme with transhydrogenase activity has not been discovered [47]. It was speculated that the redox balance mainly relied on two equilibriums: the NADPH-dependent malic enzyme YtsJ and its NAD^+^-dependent isoenzymes [47], as well as the NAD^+^-dependent glyceraldehyde-3-phosphate dehydrogenase GapA and its NADP^+^-dependent isoenzyme GapB [48]. As analyzed above, since the native systems in BSUL07 could not keep redox balance (Table 3), it was necessary to overexpress heterologous transhydrogenase to ameliorate the current redox status. Transhydrogenase from *E. coli* is well investigated and it shows good properties in metabolic engineering [16]. There are two genes *pntAB* and *udhA* encoding transhydrogenase PntAB and UdhA, respectively, in *E. coli*. Both isoforms possess divergent physiological functions: energy-dependent reduction of NADP^+^ with NADH by PntAB and reoxidation of NADPH by UdhA [16]. The experimental data showed that the redox imbalance was caused by the excess NADPH in vivo, thus UdhA was chosen for overexpression in BSUL07. The engineered strain BSUL08 obtained displayed improved redox balance (Table 3) and enhanced isobutanol production (Figure 3). Moreover, UdhA had been used to increase NADPH availability, and the productivity and yield of poly(3-hydroxybutyrate) were increased in *E.coli* [49]. The overexpression of *udhA* in a *pgi* mutant of *E. coli* improved the specific growth rate by about 25%, providing the evidence of a physiological role of UdhA [24]. Lee et al. increased the thymidine yield by employing transhydrogenase UdhA in *pgi*-disrupted strain [27]. Rxomero et al. enhanced the conversion yield of glucose into ethanol (96% of the theoretical maximum) through the heterologous expression of *udhA* [50]. Here, the overexpression of exogenous *udhA* was first carried out to enhance isobutanol production by *B. subtilis* based on a systematic analysis.

The analysis of metabolic profiling and fermentation properties of BSUL08 demonstrated that the heterologous transhydrogenase affected cell metabolism in two aspects. First, transhydrogenase could regulate intracellular redox cofactors at reasonable levels to meet the requirements of cell metabolism (Table 3). Second, the appropriate reduction of NADPH released its inhibitation on PPP and enabled the normal metabolism of down-stream glycolysis and TCA cycle to be restored (Figure 4), which guaranteed the adequate precursors and energy for cell growth and isobutanol biosynthesis.

In addition, a straightforward enginnering approach for increasing NADPH concentration for isobutanol biosynthesis was also employed in BSUL05 based on the flux analysis, and BSUL09 was obtained. The production performance, the redox and energy status of BSUL09 were then compared with other engineered strains (Figure 3, Table 3). In fact, Bastian et al. [14] and Blombach et al. [6] had reported that improving NADPH availability by overexpression of the transhydrogenase PntAB from *E. coli* is highly beneficial for isobutanol production by *E. coli* and *C. glutamicum*, respectively. These results obtained here proved the correctness of the flux analysis, and also suggested that the performance for isobutanol production by BSUL09 was poorer than that by BSUL08, but better than that by BSUL05. So, the fermentation properties of BSUL08 was investigated in a fed-batch fermentation system to further enhance the isobutanol production. During the aerobic growth phase of BSUL08, isobutanol was not formed until the exogenous acetate was almost consumed, which was in accordance with previous results obtained with PDHC-deficient strains [10]. Furthermore, the isobutanol yield was increased by 19% compared with that by BSUL05. But the yield by BSUL08 was decreased by 27% compared with that in the batch fermentation (Figure 3), and 16% by BSUL05. The reason for this behavior might be due to isobutanol toxicity to cell. One approach to solve the cytotoxicity problem and its detrimental effect on the final titer is the in situ product removeal, which was successfully applied for isobutanol fermentation [51].

In summary, the model-driven redox pathway manipulation combined with experimental validation and metabolic profiling analysis provided important insights into the potential limiting factor of improving isobutanol production by engineered isobutanol-producing strain *B. subtilis*, and this strategy can also be applied to rationally guide the titer improvement of other desired products. Besides, the obtained positive results are limited, and the experiments about using the NAD kinase or NADH kinase to generate NADPH during glycolysis and constructing of an NADH-dependent pathway for isobutanol biosynthesis using engineered enzymes, are being performed in our group and the dynamic regulatory mechanisms of *B. subtilis* response to isobutanol would be investigated further to construct a more efficient isobutanol-producing strain *B. subtilis*.

## Supporting Information

Figure S1 **Construction and confirmation of the** ***pgi*** **gene knockout plasmid.**
(DOCX)

Figure S2 **PCR confirmation of the** ***pgi*** **-deficient mutant BSUL06.**
(DOCX)

Figure S3 **Construction and confirmation of the** ***zwf*** **overexpression plasmid.**
(DOCX)

Figure S4 **PCR confirmation of the** ***zwf*** **-overexpression strain BSUL07.**
(DOCX)

Figure S5 **Construction and confirmation of the** ***udhA*** **overexpression plasmid.**
(DOCX)

Figure S6 **PCR confirmation of the** ***udhA*** **-overexpression strain BSUL08.**
(DOCX)

Table S1 **Confirmation of strains construction by enzyme assay.**
(DOCX)
