# Supplementary material for: Model-Driven Redox Pathway Manipulation for Improved Isobutanol Production in Bacillus subtilis Complemented with Experimental Validation and Metabolic Profiling Analysis
Source: PLoS One. 2014 Apr 4;9(4):e93815. doi: 10.1371/journal.pone.0093815 (PMC3976320; doi:10.1371/journal.pone.0093815)
Supplement: Figure S1 — Construction and confirmation of the pgi gene knockout plasmid. (DOCX) [file pone.0093815.s001.docx]

**Figure S1. Construction and confirmation of the *pgi* gene knockout plasmid.** Construction diagram of the gene knockout plasmid pRPCmP02 (A); Double-digestion of plasmid pRPCmP01 (*Hind*III-*Eco*RI) (B); Single-digestion of plasmid pRPCmP02 (*Hind*III) (C). M 1 kb DNA ladder; S sample, 1 the positive, 2 and 3 the negative.
