# Supplementary figures and images for: Model-Driven Redox Pathway Manipulation for Improved Isobutanol Production in Bacillus subtilis Complemented with Experimental Validation and Metabolic Profiling Analysis
Source: PLoS One. 2014 Apr 4;9(4):e93815. doi: 10.1371/journal.pone.0093815 (PMC3976320; doi:10.1371/journal.pone.0093815)

**Figure S2. PCR confirmation of the *pgi*-deficient mutant BSUL06.** M 1kb plus DNA ladder; C BSUL05; S BSUL06.


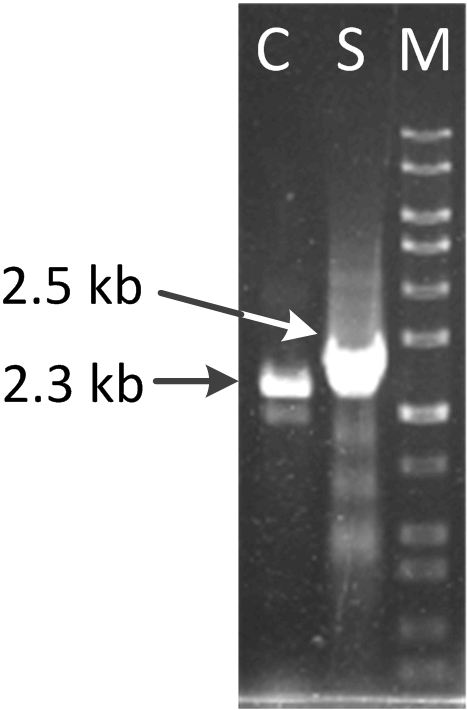

Supplement: Figure S2 — PCR confirmation of the pgi -deficient mutant BSUL06. (DOCX) [file pone.0093815.s002.docx]

**Figure S6. PCR confirmation of the *udhA*-overexpression strain BSUL08.** M 1 kb DNA ladder; S BSUL08; C BSUL07.


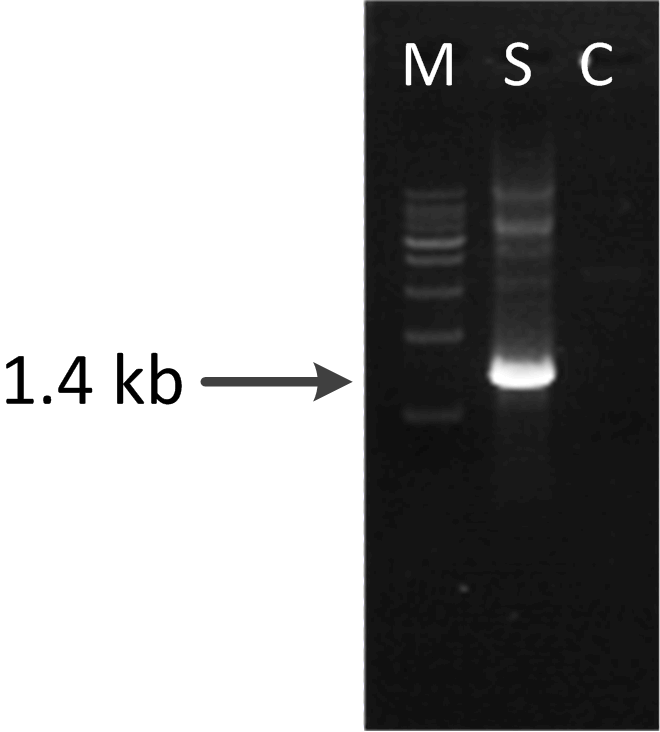

Supplement: Figure S6 — PCR confirmation of the udhA -overexpression strain BSUL08. (DOCX) [file pone.0093815.s006.docx]
