# Supplementary material for: Model-Driven Redox Pathway Manipulation for Improved Isobutanol Production in Bacillus subtilis Complemented with Experimental Validation and Metabolic Profiling Analysis
Source: PLoS One. 2014 Apr 4;9(4):e93815. doi: 10.1371/journal.pone.0093815 (PMC3976320; doi:10.1371/journal.pone.0093815)
Supplement: Figure S3 — Construction and confirmation of the zwf overexpression plasmid. (DOCX) [file pone.0093815.s003.docx]

**Figure S3. Construction and confirmation of the *zwf* overexpression plasmid.** Construction diagram of the plasmid pRPCmPZT (A); Confirmation of overlap extension of zwf and T0-T1T2 (B); PCR confirmation of plasmid pRPCmPZT (C). M1 1 kb DNA ladder; M2 Trans 5K DNA ladder; S sample, S1 *zwf*, S2 T0-T1T2, – negative control.
