# Supplementary material for: Model-Driven Redox Pathway Manipulation for Improved Isobutanol Production in Bacillus subtilis Complemented with Experimental Validation and Metabolic Profiling Analysis
Source: PLoS One. 2014 Apr 4;9(4):e93815. doi: 10.1371/journal.pone.0093815 (PMC3976320; doi:10.1371/journal.pone.0093815)

**Figure S4. PCR confirmation of the *zwf*-overexpression strain BSUL07.** M 1 kb plus DNA ladder; S BSUL07; C BSUL06.


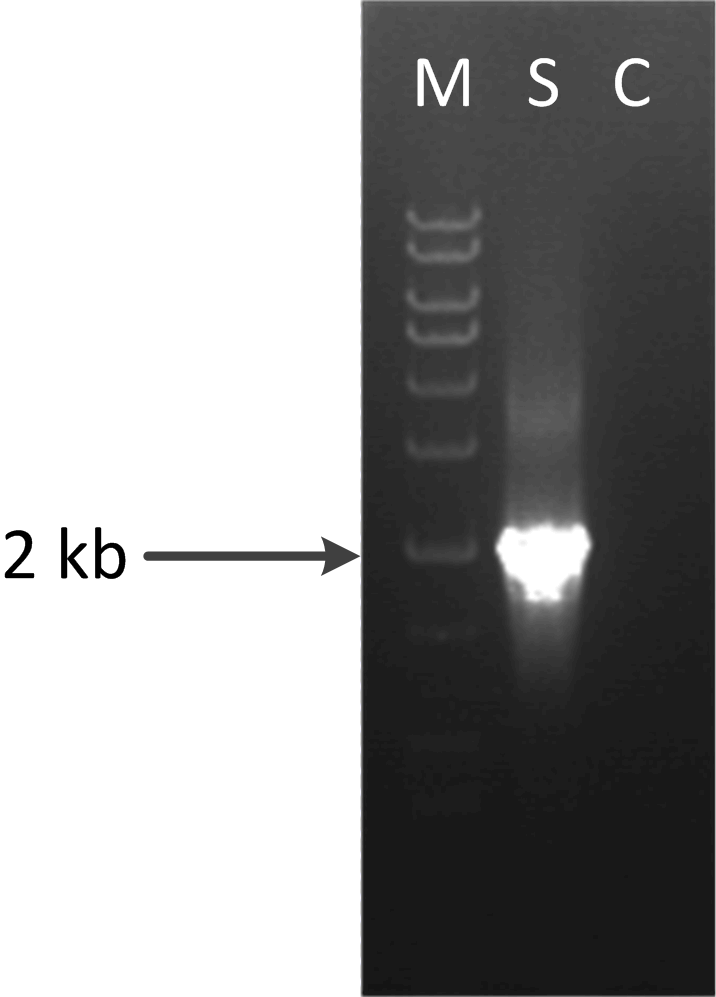

Supplement: Figure S4 — PCR confirmation of the zwf -overexpression strain BSUL07. (DOCX) [file pone.0093815.s004.docx]
