# Supplementary material for: Model-Driven Redox Pathway Manipulation for Improved Isobutanol Production in Bacillus subtilis Complemented with Experimental Validation and Metabolic Profiling Analysis
Source: PLoS One. 2014 Apr 4;9(4):e93815. doi: 10.1371/journal.pone.0093815 (PMC3976320; doi:10.1371/journal.pone.0093815)
Supplement: Figure S5 — Construction and confirmation of the udhA overexpression plasmid. (DOCX) [file pone.0093815.s005.docx]

**Figure S5. Construction and confirmation of the *udhA* overexpression plasmid.** Construction diagram of the plasmid pRPCmPZTU(A); Double-digestion confirmation of plasmid pRPCmPZTU (*Kpn*I-*BamH*I) (B). M 1 kb plus DNA ladder; S sample.
