# Supplementary material for: Model-Driven Redox Pathway Manipulation for Improved Isobutanol Production in Bacillus subtilis Complemented with Experimental Validation and Metabolic Profiling Analysis
Source: PLoS One. 2014 Apr 4;9(4):e93815. doi: 10.1371/journal.pone.0093815 (PMC3976320; doi:10.1371/journal.pone.0093815)
Supplement: Table S1 — Confirmation of strains construction by enzyme assay. (DOCX) [file pone.0093815.s007.docx]

**Table S1.** **Confirmation of strains construction by enzyme assay.**

| **Enzyme activity (U/mg)** | **BSUL05** | **BSUL06** | **BSUL07** | **BSUL08** | **BSUL09** |
| --- | --- | --- | --- | --- | --- |
| PGI | 487.6 ±22.1 | 3.2±0.1 | / | / | / |
| G6PD | / | 193.4±31.5 | 482.7±55.1 | / | / |
| UdhA | / | / | 24.7±3.2 | 103.4±14.9 | / |
| PntAB | 38.8±6.3 | / | / | / | 162.3±21.6 |
